# Assessing the impact of one million COVID-19 deaths in America: economic and life expectancy losses

**DOI:** 10.1038/s41598-023-30077-1

**Published:** 2023-02-22

**Authors:** Sachin Silva, Eric Goosby, Michael J. A. Reid

**Affiliations:** 1 Harvard TH Chan School of Public Health, Harvard University, 677 Huntington Avenue, Boston, MA 02115 USA; 2 Institute for Global Health Sciences, University of California, San Francisco, 550 16th Street, Third Floor, San Francisco, CA 94158 USA; 3 School of Medicine, University of California, San Francisco, 513 Parnassus Avenue, San Francisco, CA 94143-0410 USA

**Keywords:** Disease prevention, Health care economics, Health policy

## Abstract

Between February 2020 and May 2022, one million Americans have died of COVID-19. To determine the contribution of those deaths to all-cause mortality in terms of life expectancy reductions and the resulting economic welfare losses, we calculated their combined impact on national income growth and the added value of lives lost. We estimated that US life expectancy at birth dropped by 3.08 years due to the million COVID-19 deaths. Economic welfare losses estimated in terms of *national income growth supplemented by the value of lives lost*, was in the order of US$3.57 trillion. US$2.20 trillion of these losses were in in the non-Hispanic White population (56.50%), US$698.24 billion (19.54%) in the Hispanic population, and US$579.93 billion (16.23%) in the non-Hispanic Black population. The scale of life expectancy and welfare losses underscores the pressing need to invest in health in the US to prevent further economic shocks from future pandemic threats.

## Introduction

The COVID-19 pandemic has exposed health system frailties and exacted a terrible death toll in the United States; as of May 12, 2022, over a million Americans had died of COVID-19. Not only have those deaths been a source of both personal tragedy and social disruption, but they have also resulted in unprecedented economic losses. Because of longstanding socioeconomic inequities, these losses have not been evenly distributed. While economic analysis earlier in the pandemic estimated that COVID-19 related excess mortality may have resulted in US$4.4 trillion in losses^1^, those estimates were predicated on more conservative mortality estimates and failed to account for how the pandemic would impact specific racial and ethnic groups far more than others. In this analysis, we sought to determine life expectancy losses due to the million COVID-19 deaths and the resulting economic welfare losses, by race and ethnic origin. We used a full-income approach, which allows us to estimate the combined impact on national income growth and the value of lives lost^2^.

## Methods

Using US national life tables disaggregated by race and ethnicity^3^, we calculated life expectancies at birth and age 35, eliminating COVID-19 as a cause of death and thereby the reductions in life expectancy attributable to the million deaths due to COVID-19. We valued the economic welfare losses in three ways. First, we calculated the impact on national income growth supplemented by the value of lives lost, also known as full-income^2^, by transforming the excess hazard of COVID-19 to standardized mortality units (a 1 in 10 000 change in mortality risk), then calculating the population value of this risk change, rescaling to the life expectancy at age 35. We then multiplied by the value of a statistical life-year, calculated as a proportion of the income per capita. Secondly, we calculated the more commonly used value of a statistical life (VSL), assuming both a constant value as well as an age-dependent value. For the former, we used the value recommended by the U.S. Department of Health and Human Services (HHS) for 2022^4^. For the latter, we used age-specific 2000 VLS values^5^, which we adjusted for income and inflation assuming an income elasticity of one, adjusting upwards by the ratio of the HHS-recommended VSL for 2022^4^. Thirdly, we evaluated excess mortality risk using an age-specific VSL Year (VSLY)^6^, which we calculated by dividing the HHS-recommended VSL value by the discounted remaining life expectancy. All values were reported in 2021 US$ rates using exchange rates and deflators available from the World Bank^7^. We evaluated the sensitivity to economic parameter uncertainty and choice using a Latin hypercube sampling algorithm (Appendix). No human subjects were involved in this analysis. All analyses involved data collated from aggregated, publicly available datasets. As such, this study did not require UCSF Institutional Review Board approval.

### Human subjects research statement

No human subjects were involved in this analysis. All analyses involved data collated from aggregated, publicly available datasets. As such, this study did not require UCSF Institutional Review Board approval.

## Results

Of the one million deaths due to COVID-19, 64.5% were in the non-Hispanic White population, 16.1% in the Hispanic population, 14.3% in the non-Hispanic Black population and 3.1% in the non-Hispanic Asian population. Age-adjusted death rates from 2020 to 2021 however, were highest in the Hispanic population, followed by the non-Hispanic Black population, the non-Hispanic White population and non-Hispanic Asian population^8^. Among the non-Hispanic White population, the largest number of deaths were in the population 85 years or older. Among the Hispanic population, they were in the 65–74-year-old population.

Overall, these deaths contributed to a 3.08-year reduction in the US life expectancy at birth. At age 35, this reduction was 3.02 years. Life expectancy losses were highest in the Hispanic population (5.31 years at birth and 5.23 years at ages 35–45 years), then in the non-Hispanic Black population (3.79 years at birth and 3.75 years between the ages 35–45 years). In the non-Hispanic Asian population, 1.37 years were lost at birth and 1.24 years were lost at age 35–45 years (see Table 1).

**Table 1:** Life expectancy losses in the United States from January 1, 2020, to May 7, 2022, by race and ethnicity.

	Life Expectancy (in years)				
	Total population	non-Hispanic White	non-Hispanic Black	Hispanic	non-Hispanic Asian
At Birth					
Without COVID-19	78.85	78.78	74.78	81.85	85.56
With COVID-19	75.77	76.25	70.99	76.54	84.19
Life expectancy Loss	3.08	2.53	3.79	5.31	1.37
At Age 35					
Without COVID-19	45.50	45.32	42.43	48.31	51.47
With COVID-19	42.48	42.83	38.68	43.07	50.24
Life expectancy loss	3.02	2.49	3.75	5.23	1.24
At Age 65					
Without COVID-19	19.59	19.48	18.18	21.58	23.44
With COVID-19	17.52	17.66	15.61	17.98	22.95
Life expectancy loss	2.07	1.82	2.57	3.61	0.50

Estimated as the contribution of COVID-19 deaths towards all-cause mortality in the US life tables by Hispanic origin, race, and sex, based on age-specific death rates in 2019.

The deaths contribute to US$3.57 trillion in economic welfare losses; US$2.20 trillion of the losses were due to deaths in the non-Hispanic White population (56.5%), US$698.24 billion to deaths in the Hispanic population (19.54%), and US$579.93 billion (16.23%) to deaths in the non-Hispanic Black population. When the excess mortality was valued using the HHS-recommended VSL value of US$11.59 million (US$5.41 million–US$17.65 million), losses amounted to US$9.82 trillion. When valued using an age specific VSL value, losses amounted to US$4.85 trillion. In the former case, the non-Hispanic White population contributed US$6.17 trillion (62.87%); in the latter case, the same population contributed US$2.84 trillion (58.59%). When valued using an age-, race-, and ethnic-group-dependent VSLY, losses amounted to US$4.38 trillion, with non-Hispanic White populations contributing US$2.88 trillion (65.82%) (see Table 2).

**Table 2:** The economic value of lives lost due to COVID-19 in the United States from January 1, 2020, to May 7, 2022, by race and ethnic origin.

	Economic value of lives lost (in 2021 US$ billions)				
	Total population	non-Hispanic White	non-Hispanic Black	Hispanic	non- Hispanic Asian
In terms of Full income	$3573.89	$2018.50	$579.93	$698.24	$122.82
Assuming a constant VSL	$9821.02	$6174.79	$1406.34	$1594.97	$305.03
Assuming age dependent VSL	$4845.03	$2838.94	$760.14	$913.85	$146.27
Assuming constant VSLY	$4375.85	$2880.18	$637.55	$718.79	$139.32

Estimated in terms of full-income, assuming a constant value of a statistical life (VSL), an age-dependent VSL value and a constant value of a statistical life year (VSLY).

## Discussion

These findings highlight life expectancy reductions of a magnitude unprecedented since the 1918 influenza pandemic^9^. The million COVID-19 deaths between February 2020 and May 2022 have resulted in a drop in life expectancy at birth by 3.08 years; at age 35, by 3.02 years; and at age 65, by 2.07 years. These losses have effectively reversed all gains made in the last 40 years^10^. Reductions for Hispanic populations are twice as large as the reductions for the non-Hispanic White population. Reductions for the Black population are twice as large as the reductions for the Asian population, who had the lowest reductions. Hispanic populations had already reached the largest life expectancy reduction on record in 2020 due to early COVID-19 deaths (from 81.8 to 78.8 years). For the Black population, these early declines led to the lowest life expectancy seen since 2000 (74.7–71.8 years)^11^. COVID-19 has substantially decreased any mortality advantage for the Hispanic population had over the non-Hispanic White population, gained prior to COVID-19^11^.

Economic welfare losses measured in terms of the impact on national income growth and the value of lives lost, is in the range of US$3.7 trillion—nearly half the output losses expected from 2020 to 2030 due to COVID-19^12^. Since our estimates exclude losses due to morbidity and long-term impairment, which affects nearly half of hospitalized patients^13^, welfare losses are likely higher. The uneven distribution of these losses across racial groups is also deeply troubling. For the Hispanic population, welfare losses were proportional to their population size. For the non-Hispanic Black population however, welfare losses were approximately 30% greater compared to their population contribution. These disparities are driven by lower pre-COVID-19 life expectancies among the Black population at all ages, and are indicative of underlying health disparities, including a high prevalence of chronic conditions associated with worse COVID-19 outcomes^14^, as well as disproportionately lower socioeconomic status which is likely to lead to undesirable health outcomes^15^, and chronic stress brought on by racial discrimination^16^. Moreover, the stark disparities in life-expectancy, that existed before COVID-19 but widened during the pandemic, highlight differences in place-based risks and resource deficits experienced by Black communities in the US. Uneven distribution of primary care services^17–20^ along racial lines, for example, have been extensively documented and highlight the burden of systemic Anti-Black racism in the US healthcare system^21^.

Since the start of the pandemic, prompted by huge economic disruptions and massive public health challenges, the US government has spent US$3.7 trillion in response to COVID-19, including US$ 12.4 billion for vaccine development through Operation Warp Speed^22^. However, this investment must be weighed against the staggering welfare losses outlined in our analysis. While the current US administration had proposed an Apollo-style pandemic preparedness plan, which includes strengthening the public health system with a ‘particular focus on reducing inequities’ and will cost US $65.3 billion over the next ten years^23^, this investment maybe insufficient to address social and welfare damage resulting from COVID, especially for Hispanic and non-Hispanic Black communities that have been disproportionately impacted. While estimates of the optimal investment in pandemic preparedness vary, any set of interventions that optimize access for care for vulnerable populations and strengthens technological, surveillance, clinical, manufacturing, and regulatory capacities to respond future pandemics is likely to cost hundreds of billions of dollars^24^. Nonetheless, weighed against the full income losses outlined in this paper, such investments are likely to yield a substantial dividend. Moreover, dismantling structural racism as a key driver of shaping health inequity in the US will require coordinated and inclusive multisectoral strategies at all levels of society^25,26^. Failure to address underlying drivers of inequity is likely to have deleterious impacts on the US economy for years to come.

We acknowledge that our analysis has limitations. Estimates of full income losses are prone to VSL-related limitations such as the inconsistency in the age-VSL relationship^26^. Estimates using the age invariant VSL are inherently higher compared to estimates using an age-sensitive VSL, given the higher concentration of COVID-19 deaths in older ages. Nonetheless, our life expectancy estimated losses are consistent with prior estimates^9,27,28^, albeit higher given the million COVID-related deaths considered.

In summary, this analysis highlights the massive economic losses resulting from excess deaths from COVID-19, with Hispanic and Black Americans disproportionately impacted. The scale of welfare losses underscores the pressing need to invest in health in the United States to prevent further economic shocks from future pandemic threats.

## Supplementary Information

Supplementary Information.

## Data Availability

The datasets analyzed during current study are publicly available in CDC WONDER repository, https://wonder.cdc.gov/mcd-icd10-provisional.html.
