# Supplementary material for: Assessing the impact of one million COVID-19 deaths in America: economic and life expectancy losses
Source: Sci Rep. 2023 Feb 22;13:3065. doi: 10.1038/s41598-023-30077-1 (PMC9947095; doi:10.1038/s41598-023-30077-1)
Supplement: Supplementary file 1 — Supplementary Information. [file 41598_2023_30077_MOESM1_ESM.docx]

**Assessing the impact of one million COVID-19 Deaths in America – Economic and Life Expectancy Losses**

**Supplementary Appendix**

Sachin Silva^1,2^ Eric Goosby^2,3^, *Michael JA Reid^2,3^

1. Harvard TH Chan School of Public Health, Harvard University

677 Huntington Avenue, Boston, MA 02115, USA.

1. University of California, San Francisco, Institute for Global Health Sciences

550 16th Street, Third Floor, San Francisco, CA 94158, USA

1. University of California, San Francisco, School of Medicine

513 Parnassus Avenue, San Francisco, CA 94143-0410, USA

***Corresponding author:**

Michael JA Reid

University of California, San Francisco, Institute for Global Health Sciences

550 16th Street, Third Floor, San Francisco, CA 94158, USA

Telephone: +(1) 415-638-7332; Fax: +(1) 415-476-6953

E-mail: Michael.Reid@ucsf.edu

**Methods**

*Calculating Life Expectancies*

We calculated life expectancies at each five-year age interval, with and without the deaths due to COVID-19, using US national life tables for 2019, disaggregated by race and ethnic origin^1^. We first converted these unabridged life tables to match the age intervals at which provisional COVID-19 deaths by age, race and Hispanic origin are reported by the Center for Disease Control (CDC)^2^.

To do so, we converted the single year (_1_L_x_) values from the unabridged life tables and summed these values from ages x to x + n to calculate the total period person-years lived between ages x and x+n (_n_L_x_). We calculated the remaining values using standard life table relationships^3^. We then extracted COVID-19 deaths from February 1, 2020, to May 7, 2022, which included 998,707 deaths. Using population projections by sex, race, and Hispanic origin, through July 2022^4^, we thereafter calculated life expectancies when eliminating COVID-19 deaths^3^, for each race and ethnic group as well as the total population.

*Calculating Economic Welfare Losses*

We calculated full-income losses by transforming the excess hazard of mortality due to COVID-19 to standardized mortality units (a 1 in 10 000 change in mortality risk), then calculated the population value of this risk change, rescaling to life expectancy at age 35, and then multiplying by the value of a statistical life-year, calculated as a proportion of the income per capita^5,^ ^6^, which we extracted from the US Bureau of Economic Analysis^7^ and the Institute for Health Metrics and Evaluation^8^.

We also valued the excess mortality risk using the more commonly used value of a statistical life (VSL), using the value recommended by the U.S. Department of Health and Human Services (HHS) for 2022^9^. VSL values are generally based on estimates from working adults, based on their trade-off of wages and occupational risks. In older ages, the value of the mortality risk reduction should be less as fewer years of life expectancy remain whereas in younger ages, the value of the mortality risk reduction should be greater as more years of life expectancy remain. The use of a constant VSL value over values mortality at older ages while under valuing mortality in younger ages.

To account for this limitation, we used the age-specific values calculated by Aldy and Viscusi for 2000^10^, which we adjusted for real income^11^ and inflation (using the corresponding consumer price indices^12^), assuming an income elasticity of 1, then adjusted upwards by the ratio of the HHS recommended VSL for 2022^9^. Additionally, to account for the life expectancy experience of each racial and ethnic group, we calculated a race/ethnic group and age specific Value of a Statistical Life Year (VSLY) by dividing the HHS recommended VSL value by the discounted remaining life expectancy for the population in each ethnic group^13^. We report all values in 2021 US$ rates using exchange rates and deflator values available from the World Bank^14^.

*Uncertainty Analysis*

We conducted a probabilistic sensitivity analysis to evaluate the sensitivity of the estimates to economic parameter uncertainty and choice. We sampled 5000 draws for parameter values, from uniform distributions with parameter boundaries in Supplemental Table 1, using a Latin Hypercube Sampling algorithm^15^. Sensitivity analysis was performed using Stata (IC version 14.2) and Microsoft Excel for Mac (version 16.45).

| ***Parameter*** | ***Upper and Lower Bound*** | ***Source*** |
| --- | --- | --- |
| *US VSL* | *$5,324,706- $17,368,683* | *U.S. Department of Health and Human Services^9^* |
| *US GDP per capita* | *$57,506- $59,197* | *US Bureau of Economic Analysis^7^ and the Institute for Health Metrics and Evaluation^8^* |
| *Income elasticity* | *0.5-1.5* | *Hammitt and Robinson*^16^*.* |

**Supplemental Table 1: Upper and lower bounds of economic parameters used in the uncertainty analysis and sources.**

**
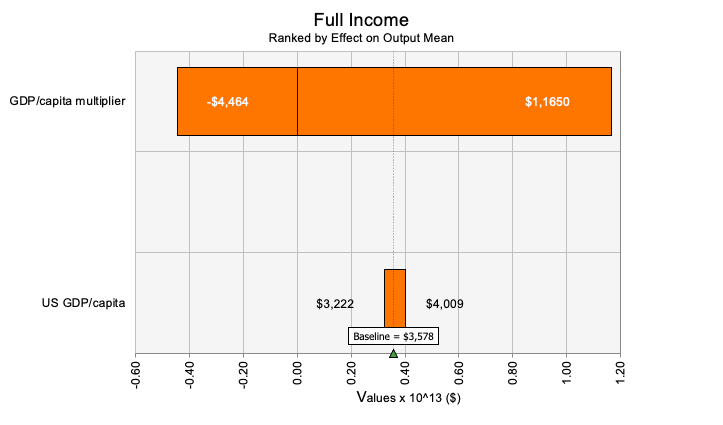
**

**(a)**

**
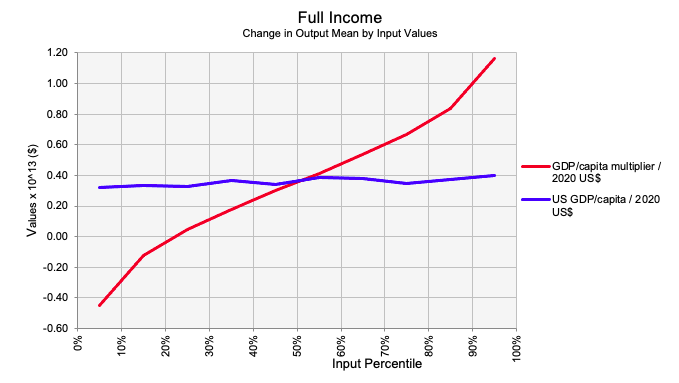
**

**(b)**

**Supplemental Figure 1:** Sensitivity of full income estimates to parameter values: (a) Tornado diagram of the sensitivity of full income estimates to GDP per capita and GDP per capita multiplier; (b) Spider diagram of the sensitivity of full income estimates to GDP per capita and GDP per capita multiplier.

**
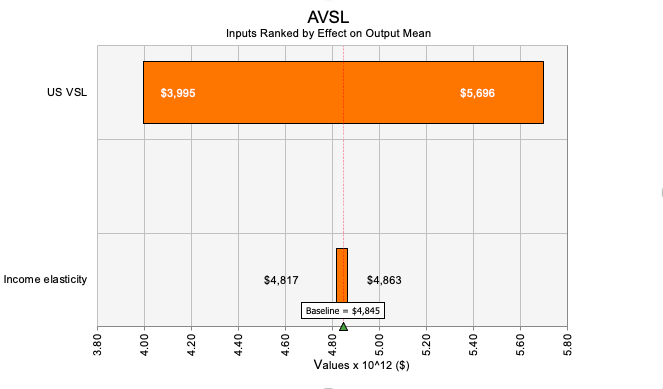
**

**(a)**

**
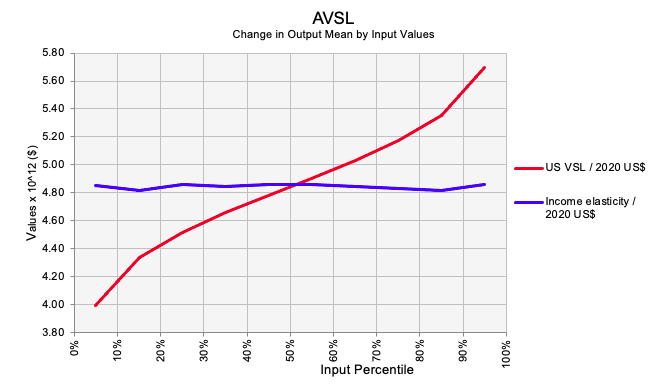
**

**(b)**

**Supplemental Figure 2:** Sensitivity of age specific VSL based estimates to parameter values: (a) Tornado diagram of the sensitivity of age specific VSL based estimates to GDP per capita and GDP per capita multiplier; (b) Spider diagram of the sensitivity of age specific VSL based estimates to GDP per capita and GDP per capita multiplier.

**Data**

| ***Age Group*** | ***Race and Ethnicity*** | ***Expected Deaths Without COVID-19*** | ***COVID-19  Deaths*** | ***Life Expectancy  Losses (Years)*** |
| --- | --- | --- | --- | --- |
| *<1* | *White* | *10,690* | *102* | *2.526* |
| *1-4* | *White* | *2,539* | *49* | *2.535* |
| *5-14* | *White* | *14,156* | *115* | *2.535* |
| *15-24* | *White* | *33,707* | *981* | *2.528* |
| *25-34* | *White* | *51,655* | *3,995* | *2.494* |
| *35-44* | *White* | *95,691* | *10,402* | *2.400* |
| *45-54* | *White* | *252,034* | *29,821* | *2.165* |
| *55-64* | *White* | *456,971* | *78,236* | *1.818* |
| *65-74* | *White* | *661,044* | *141,084* | *1.405* |
| *75-84* | *White* | *831,458* | *181,281* | *1.014* |
|  |  |  |  |  |
| ***Age  Group*** | ***Race and Ethnicity*** | ***Expected Deaths Without COVID-19*** | ***COVID-19  Deaths*** | ***Life Expectancy  Losses (Years)*** |
| *<1* | *Black* | *7,353* | *73* | *3.795* |
| *1-4* | *Black* | *1,286* | *31* | *3.831* |
| *5-14* | *Black* | *7,135* | *84* | *3.831* |
| *15-24* | *Black* | *12,822* | *646* | *3.826* |
| *25-34* | *Black* | *17,756* | *2,655* | *3.746* |
| *35-44* | *Black* | *29,720* | *6,036* | *3.530* |
| *45-54* | *Black* | *66,651* | *13,192* | *3.117* |
| *55-64* | *Black* | *91,162* | *27,753* | *2.570* |
| *65-74* | *Black* | *82,771* | *37,688* | *1.921* |
| *75-84* | *Black* | *79,247* | *31,459* | *1.273* |
|  |  |  |  |  |
| ***Age  Group*** | ***Race and Ethnicity*** | ***Expected Deaths Without COVID-19*** | ***COVID-19  Deaths*** | ***Life Expectancy  Losses (Years)*** |
| *<1* | *Hispanic* | *6,050* | *76* | *5.313* |
| *1-4* | *Hispanic* | *1,182* | *41* | *5.336* |
| *5-14* | *Hispanic* | *6,344* | *101* | *5.337* |
| *15-24* | *Hispanic* | *10,327* | *819* | *5.329* |
| *25-34* | *Hispanic* | *13,153* | *3,555* | *5.234* |
| *35-44* | *Hispanic* | *22,134* | *9,252* | *4.957* |
| *45-54* | *Hispanic* | *38,166* | *19,379* | *4.429* |
| *55-64* | *Hispanic* | *47,961* | *32,564* | *3.608* |
| *65-74* | *Hispanic* | *54,872* | *38,366* | *2.571* |
| *75-84* | *Hispanic* | *71,073* | *32,817* | *1.623* |
|  |  |  |  |  |
| ***Age  Group*** | ***Race and Ethnicity*** | ***Expected Deaths Without COVID-19*** | ***COVID-19  Deaths*** | ***Life Expectancy  Losses (Years)*** |
| *<1* | *Asian* | *3,093* | *5* | *1.371* |
| *1-4* | *Asian* | *902* | *7* | *1.375* |
| *5-14* | *Asian* | *2,119* | *11* | *1.376* |
| *15-24* | *Asian* | *600* | *81* | *1.373* |
| *25-34* | *Asian* | *4,211* | *363* | *1.238* |
| *35-44* | *Asian* | *7,554* | *794* | *1.196* |
| *45-54* | *Asian* | *6,229* | *1,844* | *1.084* |
| *55-64* | *Asian* | *40,940* | *4,184* | *0.496* |
| *65-74* | *Asian* | *134,842* | *7,418* | *0.250* |
| *75-84* | *Asian* | *385,050* | *8,063* | *0.140* |
|  |  |  |  |  |
| ***Age  Group*** | ***Race and Ethnicity*** | ***Expected Deaths Without COVID-19*** | ***COVID-19  Deaths*** | ***Life Expectancy  Losses (Years)*** |
| *<1* | *Total Population* | *27,080* | *273* | *3.080* |
| *1-4* | *Total Population* | *5,469* | *138* | *3.093* |
| *5-14* | *Total Population* | *29,891* | *333* | *3.092* |
| *15-24* | *Total Population* | *60,606* | *2,642* | *3.082* |
| *25-34* | *Total Population* | *87,191* | *11,161* | *3.020* |
| *35-44* | *Total Population* | *157,229* | *27,687* | *2.859* |
| *45-54* | *Total Population* | *373,546* | *66,556* | *2.530* |
| *55-64* | *Total Population* | *622,883* | *146,886* | *2.069* |
| *65-74* | *Total Population* | *840,672* | *229,685* | *1.544* |
| *75-84* | *Total Population* | *1,037,800* | *257,553* | *1.075* |

**Supplemental *Table 2****: Life expectancy losses in the United States from January 1, 2020, to May 7, 2022, by race and ethnicity. Estimated as the contribution of COVID-19 deaths towards all-cause mortality in the US life tables by Hispanic origin, race, and sex, based on age-specific death rates in 2019. Expected deaths without COVID-19 estimated by applying age-specific death rates (_n_M_x_) to population counts in each age interval.*

**References**

1. Arias, E. & Xu, J. United States Life Tables, 2019. *Natl Vital Stat Rep* **70**, 1–59 (2022).

2. National Center for Health Statistics. Provisional COVID-19 Deaths by Race and Hispanic Origin, and Age. *Centers for Disease Control and Prevention. Data: Provisional COVID-19 Deaths by Race and Hispanic Origin, and Age* https://data.cdc.gov/NCHS/Provisional-COVID-19-Deaths-by-Race-and-Hispanic-O/ks3g-spdg.

3. Preston, S., Heuveline, P. & Guillot, M. *Demography: Measuring and Modeling Population Processes*. (Wiley-Blackwell, 2000).

4. Bureau, U. C. 2019 Population Estimates by Age, Sex, Race and Hispanic Origin. *Census.gov* https://www.census.gov/newsroom/press-kits/2020/population-estimates-detailed.html.

5. Jamison, D. T. *et al.* Global health 2035: a world converging within a generation. *The Lancet* **382**, 1898–1955 (2013).

6. Nordhaus, W. D. Irving Fisher and the Contribution of Improved Longevity to Living Standards. *The American Journal of Economics and Sociology* **64**, 367–392 (2005).

7. U.S. Bureau of Economic Analysis. U.S. Bureau of Economic Analysis (BEA), Gross Domestic Product, Table 1. Real Gross Domestic Product and Related Measures: Percent Change from Preceding Period. https://www.bea.gov/data/gdp/gross-domestic-product.

8. Global Burden of Disease Collaborative Network. Gross Domestic Product Per Capita 1960-2050. (2021) doi:10.6069/WC70-FC19.

9. U.S. Department of Health and Human Services. Appendix D: Updating Value per Statistical Life (VSL) Estimates for Inflation and Changes in Real Income. *ASPE* https://aspe.hhs.gov/reports/updating-vsl-estimates.

10. Aldy, J. E. & Viscusi, W. K. Adjusting the Value of a Statistical Life for Age and Cohort Effects. *The Review of Economics and Statistics* **90**, 573–581 (2008).

11. U.S. Bureau of Labor Statistics. U.S. Bureau of Labor Statistics, Table 2. Median usual weekly earnings of full-time wage and salary workers, not seasonally adjusted. https://www.bls.gov/webapps/legacy/cpswktab2.htm.

12. U.S. Bureau of Labor Statistics. U.S. Bureau of Labor Statistics: Consumer Price Index (CPI) Databases. https://www.bls.gov/cpi/data.htm.

13. Robinson, L. A., Sullivan, R. & Shogren, J. F. Do the Benefits of COVID‐19 Policies Exceed the Costs? Exploring Uncertainties in the Age–VSL Relationship. *Risk Anal* 10.1111/risa.13561 (2020) doi:10.1111/risa.13561.

14. World Bank Open Data | Data. https://data.worldbank.org/?year_high_desc=false.

15. Orwa, T. O., Mbogo, R. W. & Luboobi, L. S. Uncertainty and Sensitivity Analysis Applied to an In-Host Malaria Model with Multiple Vaccine Antigens. *Int. J. Appl. Comput. Math* **5**, 73 (2019).

16. Hammitt, J. K. & Robinson, L. A. The Income Elasticity of the Value per Statistical Life: Transferring Estimates between High and Low Income Populations. *Journal of Benefit-Cost Analysis* **2**, 1–29 (2011).
